# Factors that impact dysphagia and discontinuance of oral intake in patients with progressive supranuclear palsy

**DOI:** 10.3389/fneur.2023.1259327

**Published:** 2023-09-13

**Authors:** Yuki Iwashita, George Umemoto, Shinsuke Fujioka, Hajime Arahata, Yuriko Dotsu, Asami Oike, Yoshio Tsuboi

**Affiliations:** ^1^Swallowing Disorders Center, Fukuoka University Hospital, Fukuoka, Japan; ^2^Department of Neurology, Neuro-Muscular Center, NHO Omuta National Hospital, Fukuoka, Japan; ^3^Department of Neurology, Faculty of Medicine, Fukuoka University, Fukuoka, Japan

**Keywords:** progressive supranuclear palsy, dysphagia, axial rigidity, retrocollis, cognitive decline

## Abstract

**Objective:**

To evaluate the swallowing function in the advanced stages of progressive supranuclear palsy (PSP) and clarify the factors that lead to adjustment of food consistency and discontinuation of oral intake.

**Methods:**

A total of 56 patients with PSP were recruited. Based on medical records, information about the basic attributes, clinical features (including axial rigidity and dementia), food intake, the results of a videofluoroscopic swallowing study (VFSS), and the timing of nasogastric tube transition and gastrostomy were extracted. From the VFSS images, the presence or absence of aspiration and retrocollis were assessed.

**Results:**

The average age at the onset, diagnosis, and the final follow-up examination were 67.6 ± 6.4 years, 71.6 ± 5.8 years, and 75.4 ± 5.6 years, respectively. The average duration of illness was 64.6 ± 42.8 months. Twenty-four individuals (42.9%) were continuing oral intake, while 32 were tube-fed, among whom 16 (50.0%) underwent gastrostomy tube placement. There were significant differences in the duration from the disease onset to tube feeding between the patients with and without cognitive decline at the time of the diagnosis (*p* < 0.01) and in the duration from the initial VFSS to tube feeding between the patients with and without aspiration on the initial VFSS (*p* < 0.01). There were significant differences in the duration from the diagnosis to tube feeding and from the initial VFSS to tube feeding between patients with and without axial rigidity at the time of the diagnosis (*p* < 0.05 and *p* < 0.05, respectively). Additionally, there was a significant association between axial rigidity and retrocollis (*p* < 0.01).

**Conclusion:**

Cognitive decline, axial rigidity and retrocollis, which are associated with the deterioration of dysphagia in PSP, are the highest risk factors for the discontinuation of oral intake. The early identification of these factors associated with the progression of dysphagia can contribute to the improvement of patient care and management.

## Introduction

Progressive supranuclear palsy (PSP) is a rare neurodegenerative disorder associated with the progressive bradykinesia, muscular stiffness with progressive axial dystonia, pseudobulbar palsy, dementia, and impaired voluntary eye movements. During the course of the illness, 80% of patients develop dysphagia. The duration of illness is 5–10 years, and the causes of death include aspiration pneumonia, asphyxiation, malnutrition, and frequent trauma due to falls (1).

Dysphagia symptoms appear earlier after the onset of PSP than in Parkinson’s disease (PD) (2, 3), with median latencies of 42 months reported in PSP patients and 130 months in PD patients. The most common cause of death in PSP patients is pneumonia (4) due to silent aspiration (5). Dysphagia is seen in up to 80% of PSP patients, and its early development can lead to repeated aspiration pneumonia with a short survival (1). Proposed approaches for preventing pneumonia include administering appropriate medication, adjusting the food consistency, adopting appropriate feeding techniques, and performing percutaneous endoscopic gastrostomy (PEG) feeding. Unfortunately, the response to medications in PSP patients is generally poor, with only mild improvement in dysphagia seen (6, 7), and its management in later disease stages is also challenging.

Previous studies pointed out a wide spectrum of common swallowing abnormalities in PSP patients (8), including uncoordinated lingual movements (7, 9), mastication (9, 10), oropharyngeal bolus transport (5, 9), residue in the mouth (11), delayed swallowing reflex (7, 10), impaired velar elevation (5, 9), pharyngeal residue (5, 7, 9, 10), penetration and aspiration (7, 9–11), impaired relaxation of the upper esophageal sphincter (5, 10, 12), and impaired esophageal transit (5, 9, 10). However, several clinical factors, such as limiting anterior neck flexion or progressive dementia, hamper dysphagia therapy (9).

Cognitive disability and unintelligible speech may reflect the presence of dysphagia in PSP patients and thus an increased risk of pneumonia. However, food consistency adjustment is often insufficient, with most PSP patients ultimately requiring tube feeding within a few months of developing pneumonia (13). PEG placement may prolong the survival time, but few studies have explored this notion, with no concrete confirmation yet achieved (2, 14, 15).

A previous study showed that neck stiffness was more prevalent than limb stiffness at all PSP stages (16). In this study, we therefore aimed to unearth the real process of the later stages of PSP and clarify the factors that encouraged patients and caregivers to adjust their usual food consistency and give up oral intake by analyzing clinical charts and videofluoroscopic swallowing study (VFSS) images.

## Patients and methods

### Selection of patients

We identified 127 patients with PSP admitted to the Department of Neurology at Fukuoka University Hospital and NHO Omuta Hospital between January 2009 and April 2023. Of these, we selected 56 (mean age at the disease onset, 67.6 ± 6.4 years old; 35 men, 21 women) for inclusion in the study based on the Movement Disorder Society (MDS)-PSP criteria (17) which subdivided them into PSP with Richardson’s syndrome (PSP-RS) and other PSP phenotypes (Table 1). Patients with cerebral infarction, arteriosclerotic Parkinsonism, Lewy body disease, multiple system atrophy, and corticobasal degeneration were excluded. Patients receiving end-of-life treatment for cancer, respiratory disease, or gastrointestinal disease were excluded.

**Table 1:** Patient information.

	All (*n* = 56)
Male, n (%)	35 (62.5)
Age at disease onset, years (mean ± SD)	67.6 ± 6.4
Age at diagnosis, years (mean ± SD)	71.6 ± 5.8
Age at last follow-up, years (mean ± SD)	75.4 ± 5.6
Disease duration, months (mean ± SD)	64.6 ± 42.8
PSP subtypes	
PSP-RS	12 (21.4)
PSP-P	9 (16.0)
PSP-C	5 (8.9)
PSP-PGF	4 (7.1)
PSP-CBS	2 (3.6)
PSP-SL	1 (1.8)
Not clear	23 (41)
Clinical features at diagnosis	
Fall episodes, n (%)	51 (91.1)
Axial rigidity, n (%)	45 (80.3)
Dysarthria, n (%)	39 (69.6)
Dysphagia, n (%)	33 (58.9)
Postural reflex disturbance, n (%)	46 (82.1)
Abnormal saccade or pursuit eye movements, n (%)	50 (89.3)
Cognitive decline, n (%)	45 (80.3)
Final nutritional intake status	
FOIS level ≥ 3, n (%)	24 (42.9)
FOIS level ≤ 2, n (%)	32 (57.1)
	All (*n* = 32)
PEG tube, n (%)	16 (50)

### The analysis of the disease history

**Figure 1:**
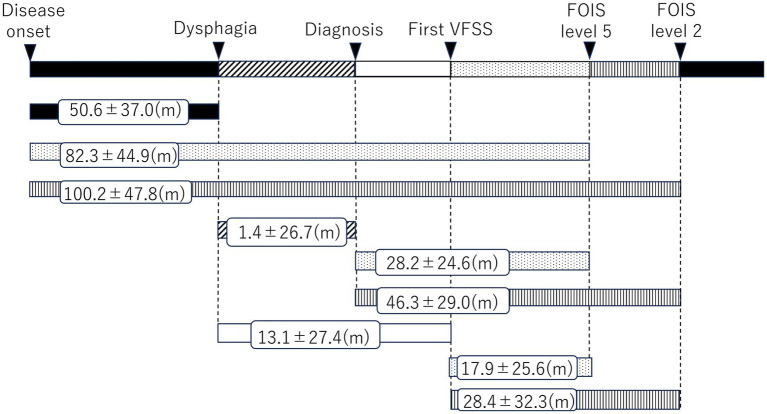
Duration between the start points and end points.

Based on the medical records of both hospitals, information about the basic attributes of each patient, including the presence or absence of dementia, food intake status, VFSS results, timing of nasogastric tube transition, and the time of gastrostomy were extracted. We defined the onset age as the age when the first symptom attributable to PSP was reported (18) (Figure 1). Regarding endpoints, the primary end point was set as the time of the definitive diagnosis, and the terminal endpoint was set as the time until discontinuation of oral intake; patients who were transferred to other hospitals or who did not see a doctor during the study period were censored at the date of their final visit. We determined the presence of cognitive decline at the diagnosis using the Mini Mental State Examination (MMSE) or Revised Hasegawa’s Dementia Scale (HDS-R). A score of < 27 on the MMSE and < 20 on the HDS-R was considered to indicate cognitive decline. The presence or absence of dysarthria at the diagnosis was determined based on their physician’s auditory evaluation in terms of articulation, fluency, and pronunciation.

This study was approved by the Ethics Committee of Fukuoka University Hospital (approval number: H21-10-001). Informed consent was obtained in the form of an opt-out provision on the study website.

### The analysis of the VFSS

For the VFSS, patients were asked to adopt the posture they used when eating meals, and fluoroscopy was performed from the side. A two-fold dilution of 110% (w/v) liquid barium was injected into the patient’s oral cavity, after which patients were signaled to start swallowing. The swallowing movements were then recorded on a DVD at 30 frames/s, and the presence of aspiration was evaluated by an assessor after the test. Patients in whom dysphagia had already been noted were first tested with 5 mL of thickened liquid and then with 5 mL of thin liquid if no aspiration was noted. Aspiration was defined as present when the liquid from the VFSS penetrated past a patient’s vocal cords into the trachea (A [+]). For patients without aspiration (A [−]), those in whom the liquid penetrated the larynx but did not reach the trachea were categorized as having laryngeal penetration (P [+]), while those without laryngeal penetration were categorized as (P [−]). A follow-up study was performed after the initial VFSS through April 2023.

### Dystonic rigidity of the neck

**Figure 2:**
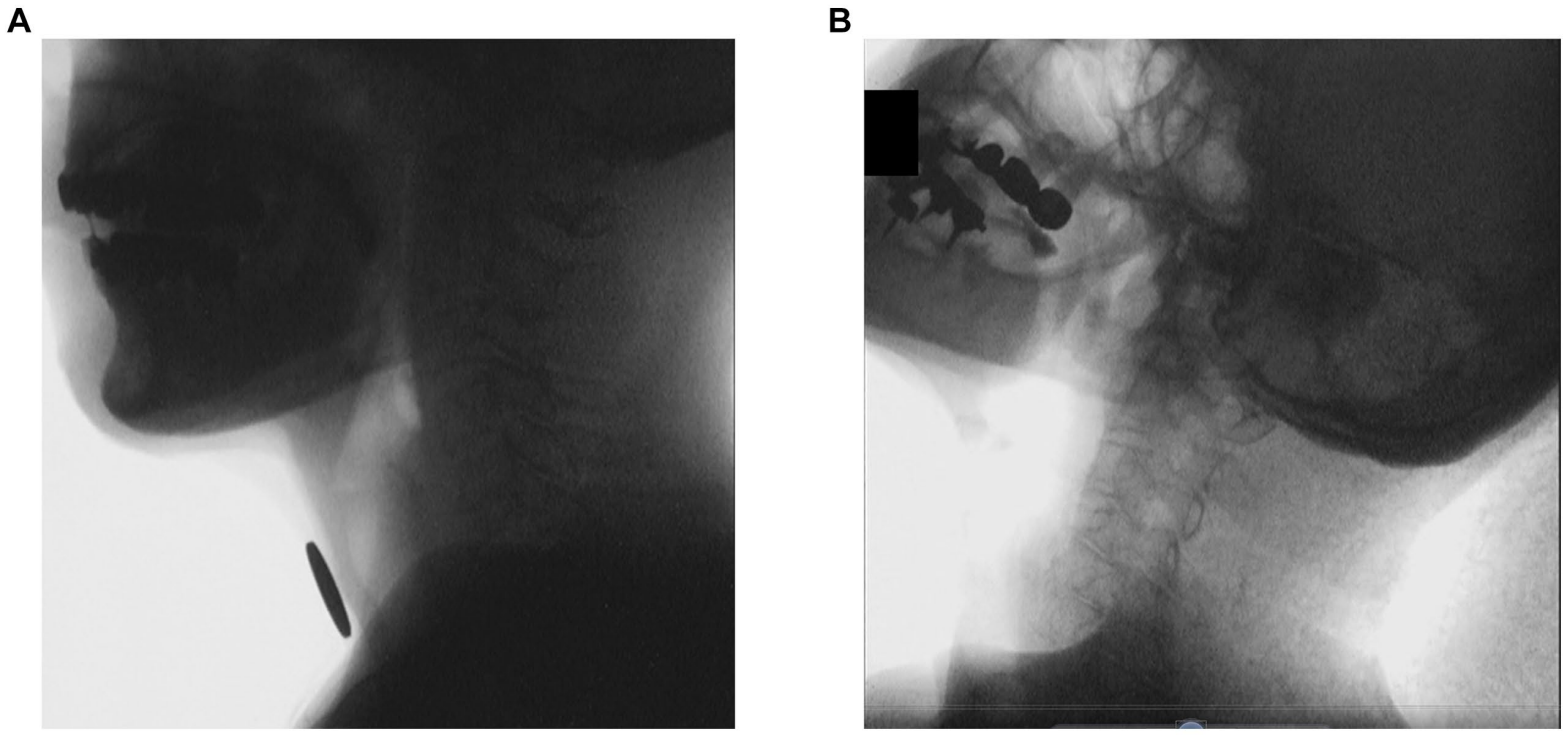
VFSS images of a representative patient with PSP at the first and last exams. **(A)** Normal neck posture at the first exam. **(B)** Retrocollis at the last exam.

Each VFSS image frame was saved in bitmap format and assessed visually. The angle changes of C1-C3 were measured in the pharyngeal phase (Figure 2), as the angles of the cervical vertebrae C1–C7 were flexed in that phase (19). Angle changes under 0.5° were taken to indicate dystonic rigidity of the neck.

### Diet type classification

The diet type was classified by a multidisciplinary team into seven levels based on the Functional Oral Intake Scale (FOIS) (20). Level 0 was tube-dependent, Level 1 could receive no oral intake, Level 2 was tube-dependent with minimal/inconsistent oral intake, Level 3 could receive consistent oral intake with some tube supplementation, Level 4 could receive total oral intake of a single consistency, Level 5 could receive total oral intake of multiple consistencies, requiring special preparation, Level 6 could receive total oral intake with no special preparation but with specific foods or liquid items avoided, and Level 7 could receive total oral intake with no restrictions.

### Data analyses

We compared clinical details, including the age at the disease onset and at death as well as the disease duration, between patient subgroups. Only patients in whom an event occurred were included in determinations of the mean results and comparisons for each milestone of advanced disease. We performed univariable analyses with Fisher’s exact test for categorical variables and a two-tailed *t*-test or the Mann–Whitney *U* test, as appropriate, for continuous variables. The interval from the onset of the first symptom to each clinical milestone or death was graphically presented using Kaplan–Meier curves, with the curves from each patient subgroup compared using a log rank test. A Cox proportional-hazards model was used to estimate the hazard ratio and 95% confidence interval.

All statistical analyses were performed using the IBM SPSS Statistics software program for Windows (version 27.0; IBM Japan, Tokyo, Japan). For all tests, *p* values of <0.05 (two-tailed) were considered statistically significant.

## Results

Among the 56 PSP patients (35 males and 21 females), the average age at the onset, diagnosis, and final follow-up examination were 67.6 ± 6.4 years, 71.6 ± 5.8 years, and 75.4 ± 5.6 years, respectively. The average duration of illness was 64.6 ± 42.8 months. The PSP subtype was identified in 35 individuals. The distribution of PSP subtypes is shown in Table 1: 12 individuals (21.4%) were diagnosed with PSP-RS and 9 individuals (16.0%) were diagnosed with PSP-P. Furthermore, among the 56 PSP patients, the following clinical symptoms were observed at the time of the definitive diagnosis: history of axial rigidity (*n* = 45; 80.3%), dysarthria (*n* = 39 individuals; 69.6%), dysphagia (*n* = 33; 58.9%), and cognitive decline (*n* = 45; 80.3%). Twenty-four individuals (42.9%) were classified as FOIS level ≥ 3, indicating primarily oral intake for nutritional management, while 32 individuals were classified as FOIS level ≤ 2, indicating nonoral intake for nutritional management. Sixteen of the 32 individuals (50.0%) underwent gastrostomy tube placement.

By setting the start points and end points, the durations were investigated. The results are presented in Figure 1. Taking disease onset as the starting point, the average duration until the onset of dysphagia was 50.6 ± 37.0 months, and the average period for transition to FOIS levels 5 and 2 (indicating the need for dysphagia diet and tube feeding) was 82.3 ± 44.9 and 100.2 ± 47.8 months, respectively. The duration from the definitive PSP diagnosis to the onset of dysphagia was 1.4 ± 26.7 months on average, indicating that dysphagia had already developed by time of the diagnosis in most cases. The period for transition to FOIS levels 5 and 2 was 28.2 ± 24.6 and 46.3 ± 29.0 months, respectively, on average. The duration from the onset of dysphagia to the initial VFSS was 13.1 ± 27.4 months on average, indicating that dysphagia had already developed at the initial VFSS in most cases. Transition to FOIS levels 5 and 2 took 17.9 ± 25.6 and 28.4 ± 32.3 months, respectively, on average.

**Figure 3:**
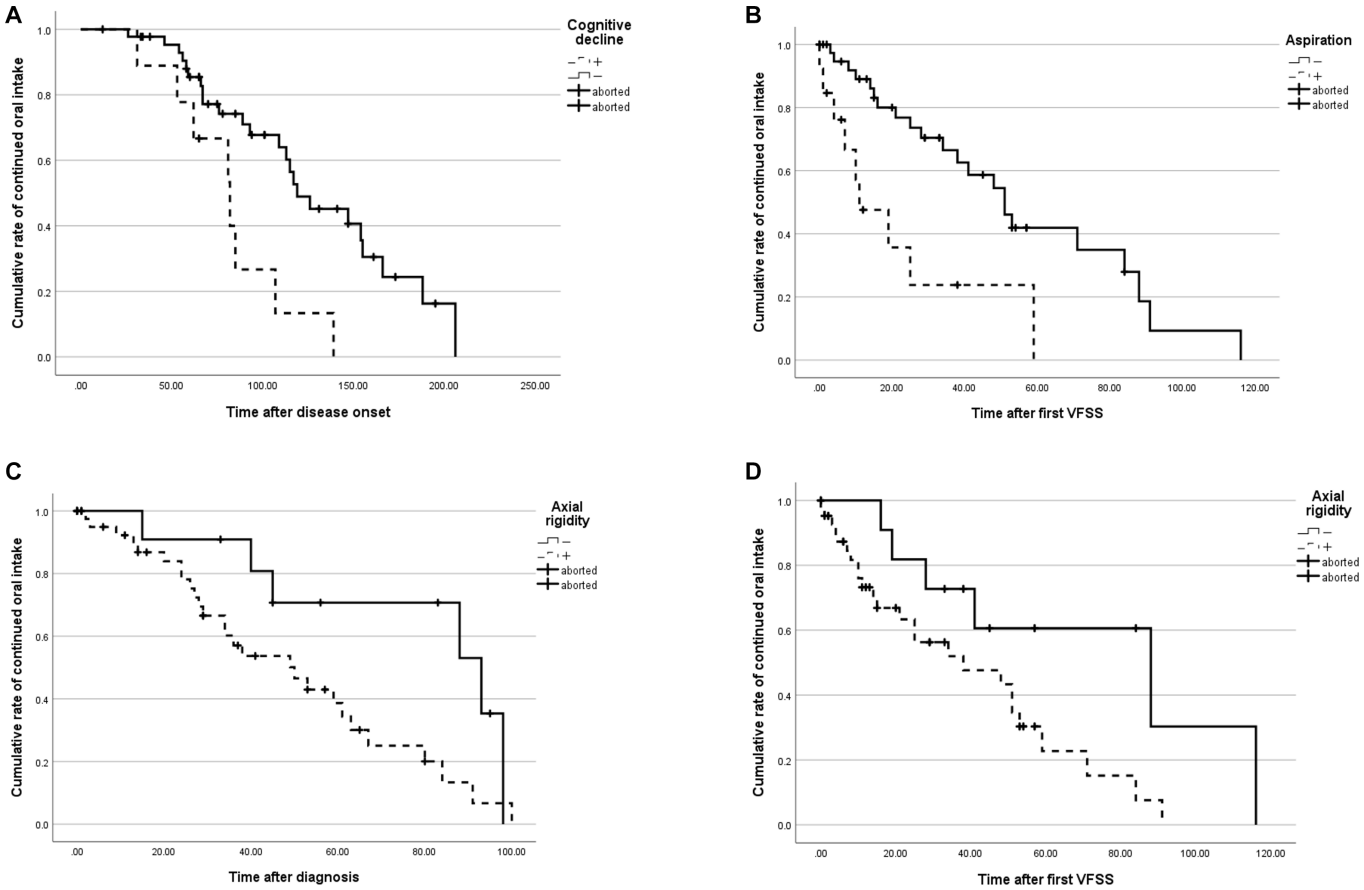
Differences in the cumulative rates of each symptom in each observation period. **(A)** Cumulative rates of continued oral intake in patients with and without cognitive decline at the diagnosis. **(B)** Cumulative rates of continued oral intake in patients with and without aspiration on the initial VFSS. **(C,D)** Cumulative rates of continued oral intake in patients with and without axial rigidity at the diagnosis.

We noted a significant difference in the duration from the disease onset to transition to FOIS level 2 between patients with and without cognitive decline at the diagnosis (*p* < 0.01, Figure 3A). In addition, a significant difference was found in the duration from the initial VFSS to transition to FOIS level 2 between patents with and without aspiration at the initial VFSS (*p* < 0.01, Figure 3B). The durations from the diagnosis to transition to FOIS level 2 and from the initial VFSS to transition to FOIS level 2 also significantly differed between patients with and without axial rigidity at the diagnosis (*p* < 0.05 and *p* < 0.05, respectively, Figures 3C,D). These significant differences were observed based on the Kaplan–Meier estimates (Table 2) and the Cox proportional hazards regression also showed differences in risk of discontinuation of oral intake. Notably, we observed no significant differences in any duration between patients with and without dysarthria at the diagnosis.

**Table 2:** Differences in cumulative rates of each symptom in each observation period.

Observation period	Start point	Disease onset			Diagnosis		First VFSS	
	End point	Dysphagia	FOIS level 5	FOIS level 2	FOIS level 5	FOIS level 2	FOIS level 5	FOIS level 2
Difference in cumulative rates of symptoms	Axial rigidity	0.12	0.87	0.18	0.15	<0.05*	0.23	<0.05*
	Dysarthria	0.12	0.08	0.06	0.21	0.10	0.75	0.95
	Cognitive decline	0.05	0.89	<0.01**	0.60	0.12	0.41	0.28
	Aspiration	0.52	0.55	0.74	0.86	0.80	0.14	<0.01**

*p < 0.05, **p < 0.01.

Additionally, there was a significant association between axial rigidity and retrocollis (*p* < 0.01, Table 3A) and between retrocollis and FOIS level ≤ 2 and tube feeding (*p* < 0.05, Table 3B). Eighteen of 35 individuals (52.4%) ultimately required tube feeding within 5.2 ± 14.3 months after the development of retrocollis. Fourteen of 31 individuals (45.2%) without retrocollis ultimately required tube feeding within 10.3 ± 12.1 months after the final VFSS.

**Table 3:** Associations between each symptom.

(A) Association between retrocollis and axial rigidity.				
		Axial rigidity		
		+	−	Total
Retrocollis	+	22	3	25
	−	23	8	31
	Total	45	11	56

(B) Association between oral and tube feeding.		
	FOIS level ≥ 3	FOIS level ≤ 2
Retrocollis (+)	7	18
Retrocollis (−)	17	14

Fisher’s exact test, *p* < 0.01 (A), *p* < 0.05 (B).

## Discussion

### Cognitive impairment

Our results showed that tube feeding was initiated earlier in patients with cognitive decline at the diagnosis than in those without such a decline. Cognitive impairment, including attention deficit, delayed information processing, difficulty in problem solving, and impaired decision making, is found at a high rate in PSP patients (21), typically progressing along with other symptoms of PSP. Cognitive decline can hamper controlling one’s intake of food and liquids, thereby increasing the risk of dysphagia (5). Patients may find it difficult to make appropriate food choices, properly prepare meals, and adjust the amount of food they consume, all of which can lead to the development of pneumonia and prevent continuation of oral intake.

### Aspiration

Our results further showed that tube feeding was also initiated earlier in patients who showed aspiration on the initial VFSS than in those without aspiration. The function of muscles involved in swallowing will deteriorate and lead to dysphagia with the progression of PSP (8). This makes it difficult to continue taking food and liquids by mouth, increasing the risk of choking and aspiration. Reduced reflexes and coordination in swallowing result in longer mealtimes and a decrease in the amount of intake, which makes nutritional management difficult (8). The rate of disease progression varies among PSP subtypes, but tube feeding typically becomes necessary within roughly 28 months after the initial VFSS or significantly earlier in patients with aspiration.

### Axial rigidity and retrocollis

The present study showed that the early initiation of tube feeding was associated with axial rigidity at the diagnosis. With the progression of PSP, the function of muscles involved in posture control deteriorates, leading to increased tension in the central axis (trunk) of the body. This makes it difficult to maintain an upright posture, resulting in forward or backward flexion (22). Progression of axial rigidity will cause stiffness and reduced control of the neck muscles. Additionally, there is a correlation between axial rigidity and retrocollis. The progression to retrocollis impairs the accuracy and efficiency of swallowing, leading to dysphagia, and makes it difficult for boluses to pass through the pharynx, in spite of any compensatory approach, modification of the consistency of food, fluids, and meal posture (23).

While axial rigidity is a major criterion for diagnosing PSP, being reported in more than half of such patients, retrocollis is not necessarily a common feature in PSP (24). Indeed, neck stiffness has consistently been reported at a might greater prevalence than a feeling of limb stiffness (16). The degree of neck rigidity was not associated with trunk and limb rigidity; however, the present study showed an association between retrocollis and axial rigidity, which leads to the discontinuation of oral intake.

### Disease milestones

A number of potential survival predictors have been proposed (25), including age at the onset (1, 18, 26, 27), early falls (26, 28), dementia (1, 28), bulbar symptoms (26), degree of disability (29), clinical phenotypes, and a short time to disability (29). Seven milestones of disease progression have been proposed: frequent falling, cognitive disability, unintelligible speech, severe dysphagia, dependence on wheelchair for mobility, use of urinary catheters, and placement in residential or nursing home care (30). However, the need for feeding tube placement is uncommon as the first event and should not be a major focus of monitoring (31). The symptoms most frequently reported at baseline were found to be motor, cognitive/behavioral, systemic and bulbar, and sleep disturbance; furthermore, neck stiffness was highly prevalent at the baseline, whereas gait impairment was relatively infrequent (16). While there does seem to be some association between certain symptoms, including dysphagia, and an increased risk of death in PSP patients, the precise symptoms that predict fatal outcomes remain controversial. As the modified PSP rating scale showed as well as this study, neck rigidity or dystonia, which can be a cause of swallowing impairment, are cited as critical factors determining prognosis (32).

### Limitation of this study

This present study was associated with some limitations. First, we could only determine the PSP subtype in 33 of the 56 participants because of the unfamiliar diagnostic criteria at the start of the study. However, these subtypes have a huge impact on the initial symptoms; for example, in PSP-P, axial rigidity is rarer than limb rigidity. The sample sizes were too small to perform an analysis according to the subtypes. Further follow-up studies with a larger population with categorization by subtype are needed. Second, we used MMSE and HDS-R to determine the presence of cognitive decline at the diagnosis. However, these two tools are merely screening methods for evaluating cognitive dysfunction and are not always suitable for evaluating cognitive dysfunction in PSP, especially to differentiate PSP-CBS from PSP-RS. As a practical matter, they are widely accepted by physicians in daily clinical practice for PSP as a screening tool to recognize moderate cognitive impairment despite variations in sensitivity and specificity (33, 34); we therefore adopted the test results. Third, in this study, we could not track some participants until the terminal phase because of difficulties keeping in contact with them or their family due to changing hospitals or switching to residential or nursing home care. The number of aborted cases during follow-up could influence the presentation of the Kaplan–Meier curves. In the future, a new tracking system should be introduced for patients in the terminal phase of PSP. Despite these limitations, these results will contribute to the understanding of the disease and the improvement of treatment strategies. Understanding the predictive factors and complications associated with dysphagia progression will aid in managing and caring for patients.

## Conclusion

In the present study, we analyzed information on the progression of dysphagia in patients with PSP. Cognitive decline, axial rigidity, and retrocollis exacerbate dysphagia in PSP and are the strongest risk factors for discontinuation of oral intake. Identifying such factors as early as possible may help improve patient care and management. Future research is expected to lead to the elucidation of specific predictive factors and the development of treatment strategies.

## Data availability statement

The original contributions presented in the study are included in the article/supplementary material, further inquiries can be directed to the corresponding author.

## Ethics statement

The studies involving humans were approved by the Ethics Committee of Fukuoka University Hospital. The studies were conducted in accordance with the local legislation and institutional requirements. The ethics committee/institutional review board waived the requirement of written informed consent for participation from the participants or the participants’ legal guardians/next of kin because informed consent was obtained in the form of an opt-out provision on the study website.

## Author contributions

YI: Formal Analysis, Writing – original draft, Writing – review & editing, Data curation, Investigation. GU: Formal Analysis, Writing – original draft, Writing – review & editing, Conceptualization, Funding acquisition, Methodology, Project administration, Resources, Supervision. SF: Supervision, Writing – review & editing. HA: Data curation, Investigation, Writing – review & editing. YD: Data curation, Investigation, Writing – review & editing. AO: Data curation, Investigation, Writing – review & editing. YT: Funding acquisition, Resources, Supervision, Writing – review & editing.

## Funding

The author(s) declare financial support was received for the research, authorship, and/or publication of this article. This work was financially supported by JSPS KAKENHI Grant number (21K10034) and by funding from Fukuoka University (216007).

## Conflict of interest

The authors declare that the research was conducted in the absence of any commercial or financial relationships that could be construed as a potential conflict of interest.

## Publisher’s note

All claims expressed in this article are solely those of the authors and do not necessarily represent those of their affiliated organizations, or those of the publisher, the editors and the reviewers. Any product that may be evaluated in this article, or claim that may be made by its manufacturer, is not guaranteed or endorsed by the publisher.
